# Supplementary material for: Hepatoprotective Activity of Ethanol Extract of Rice Solid-State Fermentation of Ganoderma tsugae against CCl4-Induced Acute Liver Injury in Mice
Source: Molecules. 2022 Aug 22;27(16):5347. doi: 10.3390/molecules27165347 (PMC9416711; doi:10.3390/molecules27165347)
Supplement: Supplementary file 1 [file molecules-27-05347-s001.zip › molecules-1857010-supplementary/Supplementary/Table S1.pdf]

Table S1. The results of response surface optimization test for G42 rice solid fermentation.

Number	Factor and level			Triterpene content （mg/g）
	A Temperature （℃）	B Time （d）	C Inoculation amount （mL）	
1	25	40	30	45.47
2	30	40	30	49.59
3	25	50	30	42.75
4	30	50	30	47.36
5	25	45	20	45.71
6	30	45	20	49.68
7	25	45	25	38.06
8	30	45	25	40.32
9	27	40	20	47.62
10	27	50	20	46.26
11	27	40	25	39.22
12	27	50	30	38.84
13	27	45	25	50.86
14	27	45	25	51.21
15	27	45	25	51.79
16	27	45	25	50.92
17	27	45	50	50.56
