# Supplementary material for: Hepatoprotective Activity of Ethanol Extract of Rice Solid-State Fermentation of Ganoderma tsugae against CCl4-Induced Acute Liver Injury in Mice
Source: Molecules. 2022 Aug 22;27(16):5347. doi: 10.3390/molecules27165347 (PMC9416711; doi:10.3390/molecules27165347)
Supplement: Supplementary file 1 [file molecules-27-05347-s001.zip › molecules-1857010-supplementary/Supplementary/Table S2.pdf]

Table S2. Differential compounds of rice before and after G42 rice solid fermentation.

No.	RT/min	Component name	Formula	Theoretical mass	[M-H]-	ppm	MS/MS Fragments[2]
1	2.43	Beta-ethoxyethyl sebacate	$C_{18}H_{34}O_6$	346.2346	345.2273	-2.7	229.1434,211.1330,183.1385
2	2.71	10-[2-(2-Butoxyethoxy)ethoxy]-10-oxodecanoic acid	$C_{18}H_{34}O_6$	346.2346	345.2273	-2.8	229.1441,211.1337,171.1018
3	8.67	(10E,15Z)-9,12,13-Trihydroxy-10,15-octadecadienoic acid	$C_{18}H_{32}O_5$	328.2241	327.2168	-2.6	229.1446,211.1328,171.1012
4	12.41	9,10,13-Trihydroxy-11-octadecenoic acid	$C_{18}H_{34}O_5$	330.24	329.2327	-2	311.2225,211.1331,183.1384
5	12.79	(9Z)-12,13,17-Trihydroxy-9-octadecenoic acid	$C_{18}H_{34}O_5$	330.2399	329.2326	-2.3	171.1018,139.1120,137.0966
6	13.09	Sanleng acid	$C_{18}H_{34}O_5$	330.24	329.2327	-2	311.2209,229.1440,171.1019
7	15.85	Sanleng acid isomer	$C_{18}H_{34}O_5$	330.2398	329.2325	-2.6	201.1129,171.1016
8	16.75	9,10,13-Trihydroxy-11-octadecenoic acid isomer	$C_{18}H_{34}O_5$	330.2398	329.2325	-2.5	201.1125,171.1018
9	17.46	(-)-pinellic acid isomer	$C_{18}H_{34}O_5$	330.2398	329.2325	-2.5	201.1122,171.1019,127.1120
10	17.81	Platycodigenin	$C_{30}H_{48}O_7$	520.3398	519.3325	-0.4	501.3222,322.2145,26

							1.1481
11	18.58	Cucurbitacin IIb	$C_{30}H_{48}O_7$	520.3399	519.3326	-0.2	501.3221,322.2140,101.0600
12	22.59	Ganolucidic acid B	$C_{30}H_{46}O_6$	502.3291	501.3219	-0.6	483.3120,330.2202,137.0604
13	24.07	(5R,6S,7R)-5-Amino-2,4,6,7-tetrahydroxy-3-henicosanone	$C_{21}H_{43}NO_5$	389.3134	388.3062	-1.8	267.2322,255.2322,225.2215
14	24.68	O-(Hydroxy{(2R)-2-hydroxy-3-[(2-methoxy-14-methylpentadecyl)oxy]propoxy}phosphoryl)-L-serine isomer	$C_{23}H_{48}NO_9P$	513.3068	512.2996	0.3	452.2775,239.2014,227.2009
15	24.89	9,10-DiHODE	$C_{18}H_{32}O_4$	312.2294	311.2222	-2	295.2268,277.2170,109.0651
16	25.01	UNKONWN	$C_{23}H_{53}N_5O_{11}P_2$	637.3231	636.3158	2.2	
17	25.08	1-Myristoyl-2-hydroxy-sn-glycero-3-PE	$C_{19}H_{40}NO_7P$	425.2542	424.247	0	227.2011,196.0378,140.0109
18	25.18	(9Z)-2-Hydroxy-17-oxo-9-octadecenoic acid	$C_{18}H_{32}O_4$	312.2296	311.2224	-1.3	295.2265,279.2325,239.2007
19	25.24	O-(Hydroxy{(2R)-2-hydroxy-3-[(2-methoxy-14-methylpentadecyl)oxy]propoxy}phosphoryl)-L-serine	$C_{23}H_{48}NO_9P$	513.3069	512.2996	0.4	452.2785,363.1937,227.2009
20	25.37	(11E,13E)-9,10-dihydroxy-11,13-octadecadienoic acid isomer-2	$C_{18}H_{32}O_4$	312.2295	311.2222	-1.8	171.1014,139.1116
21	25.63	(2R)-1-{[(2-Aminoethoxy)(hydroxy)phosphoryl]oxy}-3-hydroxy-2-propanyl (9Z,12Z,15	$C_{23}H_{42}NO_7P$	475.2701	474.2628	0.5	277.2168,152.9952,78

		Z)-9,12,15-octadecatrienoate					.9583
22	25.78	(2S)-2-Amino-3-[(hydroxy{[(2R,3R)-2-{[(9Z)-9-octadecenoyloxy]methyl}tetrahydro-2H-pyran-3-yl]oxy}phosphoryl)oxy]propanoic acid (non-preferred name)	$C_{27}H_{50}NO_9P$	563.3222	562.315	-0.2	502.2946,277.2168,78.9584
23	25.83	1-Deoxy-1-{[(7R,18Z,21Z)-4,7-dihydroxy-4-oxido-10-oxo-3,5,9-trioxa-4lambda~5~-phosphaheptacosa-18,21-dien-1-yl]amino}-beta-D-fructopyranose	$C_{29}H_{54}NO_{12}P$	639.339	638.3317	0.9	476.2789,279.2323,152.9953
24	26.31	1-Deoxy-1-{[(7R,17Z,20Z)-4-hydroxy-7-(hydroxymethyl)-4-oxido-9-oxo-3,5,8-trioxa-4lambda~5~-phosphahexacosa-17,20-dien-1-yl]amino}-beta-D-fructopyranose	$C_{29}H_{54}NO_{12}P$	639.3388	638.3315	0.7	476.2784,279.2324,152.9952
25	26.55	3-{[(2-Aminoethoxy)(hydroxy)phosphoryl]oxy}-2-hydroxypropyl (9Z,12Z)-9,12-octadecadienoate	$C_{23}H_{44}NO_7P$	477.2855	476.2782	-0.1	417.2384,279.2322,152.9950
26	26.57	1-Deoxy-1-{[(7R)-4,7-dihydroxy-4-oxido-10-oxo-3,5,9-trioxa-4lambda~5~-phosphapentacos-1-yl]amino}-beta-D-fructopyranose	$C_{27}H_{54}NO_{12}P$	615.3385	614.3312	0.2	452.2778,255.2321,152.9950
27	26.7	1-[2-(4-{(E)-[4-(2-{[3-({4-[(3-Aminopropyl)amino]butyl}amino)propyl]amino}ethoxy)phenyl]diazenyl}phenoxy)ethyl]-1H-pyrrole-2,5-dione	$C_{30}H_{43}N_7O_4$	565.3381	564.3308	0.8	483.3110,279.2323
28	27	1-{[(2-Aminoethoxy)(hydroxy)phosphoryl]oxy}-3-hydroxy-2-propanyl (4Z,7Z)-4,7-octadecadienoate	$C_{23}H_{44}NO_7P$	477.2857	476.2784	0.3	433.2354,279.2324,196.0373
29	27.11	1-Deoxy-1-{[(7R)-4-hydroxy-7-(hydroxymethyl)-4-oxido-9-oxo-3,5,8-trioxa-4lambda~5~-phosphatetracos-1-yl]amino}-beta-D-fructopyranose	$C_{27}H_{54}NO_{12}P$	615.3389	614.3316	0.8	452.2785,255.2324,196.0373
30	27.15	1-[(9Z,12Z)-heptadecadienoyl]-sn-glycero-3-phosphocholine	$C_{25}H_{48}NO_7P$	505.3170	504.3097	0.3	279.2324,224.0688,78.9583
31	27.15	UNKONWN	$C_{27}H_{52}NO_9P$	565.3381	564.3309	0.3	

32	27.22	1-linoleoyl-sn-glycero-3-phospho-D-myo-inositol	$C_{27}H_{49}O_{12}P$	596.2965	595.2893	0.6	333.0578,315.0480,241.0114
33	27.35	1-Palmitoyl-2-hydroxy-sn-glycero-3-PE	$C_{21}H_{44}NO_7P$	453.2856	452.2783	0.1	281.2474,255.2323,196.0373
34	27.88	2-hexadecanoyl-sn-glycero-3-phosphoethanolamine	$C_{16}H_{41}N_9O_2P_2$	453.2854	452.2781	-1	281.2479,255.2323,78.9583
35	28.02	O-(Hydroxy{(2R)-2-hydroxy-3-[(2-methoxyoctadecyl)oxy]propoxy}phosphoryl)-L-serine isomer	$C_{25}H_{52}NO_9P$	541.3381	540.3308	0.2	480.3098,255.2325,224.0687
36	28.04	1-pentadecanoyl-sn-glycero-3-phosphocholine	$C_{23}H_{48}NO_7P$	481.3167	480.3095	-0.2	255.2323,224.0686,78.9583
37	28.22	(2R)-2-Hydroxy-3-[(hydroxy{[(1S,2R,3R,4S,5S,6R)-2,3,4,5,6-pentahydroxycyclohexyl]oxy}phosphoryl)oxy]propyl palmitate	$C_{25}H_{49}O_{12}P$	572.2960	571.2887	-0.3	391.2249,281.2479,152.9951
38	28.35	9-HODE	$C_{18}H_{32}O_3$	296.2343	295.2270	-2.8	277.2164,195.1376,113.0967
39	28.37	O-{[(2R)-3-(Henicosanoyloxy)-2-hydroxypropoxy](hydroxy)phosphoryl}-L-serine	$C_{27}H_{54}NO_9P$	567.3533	566.3460	-0.5	506.3250,281.2478,168.0425
40	28.68	2-Aminoethyl 2-hydroxy-3-{[(9E)-1-oxonio-9-octadecen-1-yl]oxy}propyl phosphate	$C_{23}H_{46}NO_7P$	479.3013	478.2940	0.2	281.2479,140.0110,78.9583
41	28.84	(2R)-2-Carboxy-2-(trimethylammonio)ethyl 2-methoxy-3-[(12-oxoheptadecyl)oxy]propyl phosphate	$C_{27}H_{54}NO_9P$	567.3536	566.3463	-0.1	506.3256,281.2480,78.9583

42	29.29	1-oleoyl-sn-glycero-3-phospho-D-myo-inositol	$C_{27}H_{51}O_{12}P$	598.3115	597.3043	-0.4	281.2478,241.0112,96.9687
43	29.37	3-{[(2,3-Dihydroxypropoxy)(hydroxy)phosphoryl]oxy}-2-hydroxypropyl palmitate	$C_{22}H_{45}O_9P$	484.2799	483.2726	-0.4	255.2322,152.9951,78.9583
44	29.92	(2R)-3-({[(2S)-2,3-Dihydroxypropoxy](hydroxy)phosphoryl}oxy)-2-hydroxypropyl palmitate	$C_{22}H_{45}O_9P$	484.2802	483.2729	0.1	255.2326,152.9952.78.9583
45	31.48	1-(9Z-octadecenoyl)-sn-glycero-3-phospho-(1'-sn-glycerol)	$C_{24}H_{47}O_9P$	510.2958	509.2885	0	281.2479,152.9952,78.9583
46	32.85	Benzenepropanoic acid, 3-(1,1-dimethylethyl)-4-hydroxy-5-methyl-, 1,2-ethanediylbis(oxy-2,1-ethanediyl) ester	$C_{34}H_{50}O_8$	586.3506	585.3434	0.1	367.2121,233.1024,149.0814
47	33.05	(3alpha,5xi,12alpha,25S)-3-{[(3S)-4-Carboxy-3-hydroxy-3-methylbutanoyl]oxy}-12-hydroxy-24-methylenelanost-8-en-26-oic acid	$C_{37}H_{58}O_8$	630.4140	629.4068	1.4	485.3643,441.3740,101.0238
48	33.3	Hexadecaneperoxoic acid	$C_{16}H_{32}O_3$	272.2349	271.2276	-1	225.2220,101.0237,57.0338
49	34.28	(3S)-3-Hydroxy-5-{[(3alpha,12alpha,23S)-12-hydroxy-24-methyl-26-oxo-23,26-epoxylanosta-8,24-dien-3-yl]oxy}-3-methyl-5-oxopentanoic acid	$C_{37}H_{56}O_8$	628.3982	627.3909	1.1	565.3902,525.3588,329.2482
50	34.46	(3beta,12alpha,25S)-3-{[(3S)-4-Carboxy-3-hydroxy-3-methylbutanoyl]oxy}-12-hydroxy-24-methylenelanost-8-en-26-oic acid	$C_{37}H_{58}O_8$	630.4139	629.4066	1.1	527.3743,441.3739,81.0338
51	34.66	(3beta,12alpha,25S)-3-{[(3S)-4-Carboxy-3-hydroxy-3-methylbutanoyl]oxy}-12-hydroxy-24-methylenelanost-8-en-26-oic acid isomer	$C_{37}H_{58}O_8$	630.4137	629.4064	0.8	567.4060,441.3739,81.0338

52	34.76	Methyl 3-acetoxy-27-hydroxylup-20(29)-en-28-oate	$C_{33}H_{52}O_5$	528.3817	527.3744	0.4	465.3371,279.2324,277.2164
53	36.19	(3alpha,5xi)-3-[(4-Carboxy-3-hydroxy-3-methylbutanoyl)oxy]-24-methylenelanost-8-en-21-oic acid	$C_{37}H_{58}O_7$	614.4186	613.4114	0.6	551.4104,469.3687,101.0236
54	36.35	18-hydroxystearic acid	$C_{18}H_{36}O_3$	300.266	299.2587	-1.6	253.2531,251.2372,225.2209
55	36.83	(3alpha,12alpha,25S)-12-Hydroxy-3-[(3-hydroxy-5-methoxy-3-methyl-5-oxopentanoyl)oxy]-24-methylenelanost-8-en-26-oic acid isomer	$C_{38}H_{60}O_8$	644.4297	643.4224	1.3	525.3954,441.3741,83.0131
56	38.72	Methyl 2-(hydroxymethyl)octadecanoate	$C_{20}H_{40}O_3$	328.2970	327.2898	-2.1	281.2842,277.2529,253.2528
