# Supplementary material for: Hepatoprotective Activity of Ethanol Extract of Rice Solid-State Fermentation of Ganoderma tsugae against CCl4-Induced Acute Liver Injury in Mice
Source: Molecules. 2022 Aug 22;27(16):5347. doi: 10.3390/molecules27165347 (PMC9416711; doi:10.3390/molecules27165347)
Supplement: Supplementary file 1 [file molecules-27-05347-s001.zip › molecules-1857010-supplementary/Supplementary/Table S3.pdf]

Table S3. The information of *Ganoderma* spp. strains used in this study

Number	Strain name	Source	Type	Name
G10	CCMJ2444	Heilongjiang China	Wild	*Ganoderma tsugae*
G12	CCMJ2446	Guizhou China	Wild	*Ganoderma tsugae*
G17	CCMJ2451	Russia	Cultivated	*Ganoderma tsugae*
G20	CCMJ2454	Jilin China	Cultivated	*Ganoderma tsugae*
G27	CCMJ2461	Heilongjiang China	Cultivated	*Ganoderma tsugae*
G40	CCMJ2474	Heilongjiang China	Wild	*Ganoderma tsugae*
G41	CCMJ2475	Jilin China	Wild	*Ganoderma tsugae*
G42	CCMJ2476	Jilin China	Wild	*Ganoderma tsugae*
G43	CCMJ2477	Jilin China	Cultivated	*Ganoderma tsugae*
G78	CCMJ3025	Zhejiang China	Cultivated	*Ganoderma sichuanense*
