# Supplementary figures and images for: Hepatoprotective Activity of Ethanol Extract of Rice Solid-State Fermentation of Ganoderma tsugae against CCl4-Induced Acute Liver Injury in Mice
Source: Molecules. 2022 Aug 22;27(16):5347. doi: 10.3390/molecules27165347 (PMC9416711; doi:10.3390/molecules27165347)

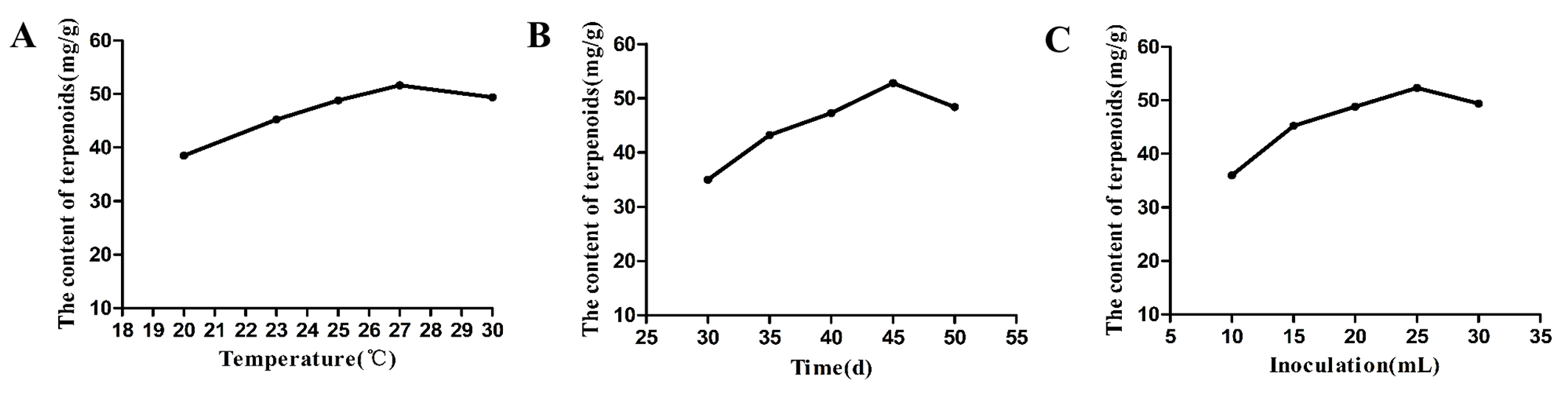

Supplement: Supplementary file 1 [file molecules-27-05347-s001.zip › molecules-1857010-supplementary/Supplementary/Figure S1.tif]

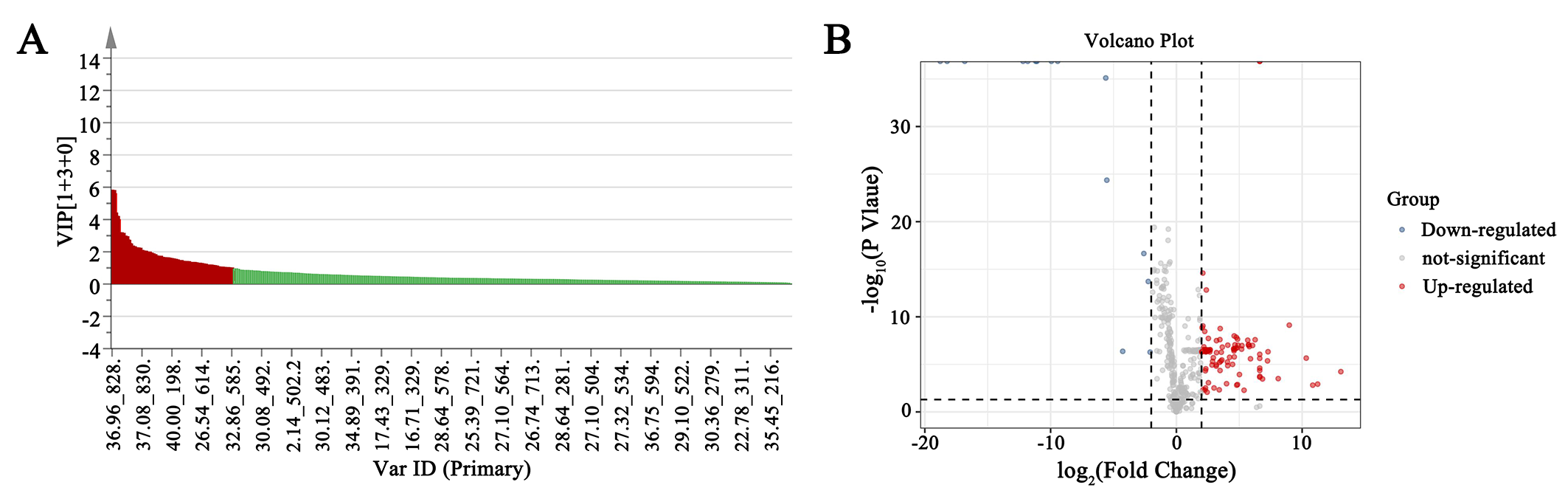

Supplement: Supplementary file 1 [file molecules-27-05347-s001.zip › molecules-1857010-supplementary/Supplementary/Figure S2.tif]

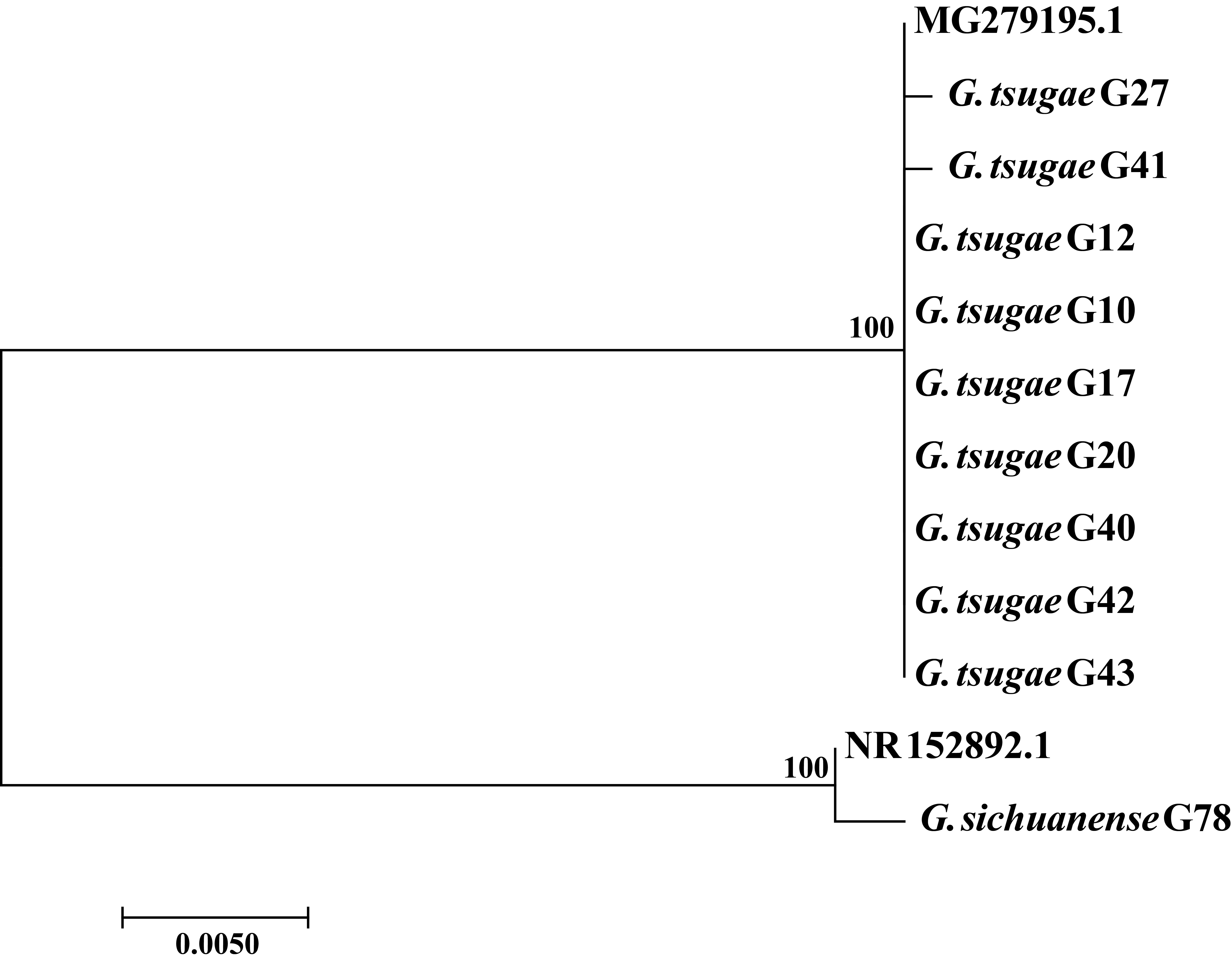

Supplement: Supplementary file 1 [file molecules-27-05347-s001.zip › molecules-1857010-supplementary/Supplementary/Figure S3.tif]
